# Synaptic Hyaluronan Synthesis and CD44-Mediated Signaling Coordinate Neural Circuit Development

**DOI:** 10.3390/cells10102574

**Published:** 2021-09-28

**Authors:** Emily S. Wilson, Karen Litwa

**Affiliations:** Department of Anatomy and Cell Biology, Brody School of Medicine, East Carolina University, Greenville, NC 27834, USA; wilsonemi21@ecu.edu

**Keywords:** induced pluripotent stem cells, synapse, hyaluronan, rhogtpases

## Abstract

The hyaluronan-based extracellular matrix is expressed throughout nervous system development and is well-known for the formation of perineuronal nets around inhibitory interneurons. Since perineuronal nets form postnatally, the role of hyaluronan in the initial formation of neural circuits remains unclear. Neural circuits emerge from the coordinated electrochemical signaling of excitatory and inhibitory synapses. Hyaluronan localizes to the synaptic cleft of developing excitatory synapses in both human cortical spheroids and the neonatal mouse brain and is diminished in the adult mouse brain. Given this developmental-specific synaptic localization, we sought to determine the mechanisms that regulate hyaluronan synthesis and signaling during synapse formation. We demonstrate that hyaluronan synthase-2, HAS2, is sufficient to increase hyaluronan levels in developing neural circuits of human cortical spheroids. This increased hyaluronan production reduces excitatory synaptogenesis, promotes inhibitory synaptogenesis, and suppresses action potential formation. The hyaluronan receptor, CD44, promotes hyaluronan retention and suppresses excitatory synaptogenesis through regulation of RhoGTPase signaling. Our results reveal mechanisms of hyaluronan synthesis, retention, and signaling in developing neural circuits, shedding light on how disease-associated hyaluronan alterations can contribute to synaptic defects.

## 1. Introduction

The majority of neurodevelopmental disorders exhibit synaptic alterations [1,2]. These synaptic alterations disrupt the balance between excitation and inhibition of developing neural circuits, often leading to hyperexcitability [3,4,5]. For example, reductions in the brain extracellular matrix glycosaminoglycan, hyaluronan (HA), increase the excitability of neural networks and drive the formation of epileptic seizures [6]. In order to elucidate the role of HA in the emergence of neural circuit disruption, our research focuses on the mechanisms of synapse formation [7,8,9,10,11,12]. In humans, synapse formation (or synaptogenesis) begins in mid-fetal gestation [12,13]. In contrast to previous studies suggesting that HA is absent from synapses [14,15], our recent work demonstrates that HA is present in the cleft between the pre-and post-synaptic compartments of developing excitatory synapses [11]. To determine whether HA’s synaptic localization is developmental specific, our current study confirms that HA is present at the cleft of newly formed synapses of the mouse neonatal brain, but is reduced in the cleft of mature synapses and increases in regions surrounding the synapse, consistent with previous observations of a peri-synaptic extracellular matrix [16,17]. This shift coincides with the formation of perineuronal nets around inhibitory neurons, which form postnatally and are prominent in the adult brain [18]. PNNs signal the closure of developmental plasticity and maintain the established balance between inhibitory and excitatory signaling [19], with the absence of PNNs leading to increased neural network excitability [20]. Prior to PNN formation, our work establishes that HA protects the developing brain from the emergence of hyperexcitability by suppressing excitatory synaptogenesis [11]. However, the mechanisms of HA synthesis and signaling within developing neural circuits have yet to be elucidated.

HA is synthesized by three hyaluronan synthases (HAS 1–3). Mammalian HASs consist of seven transmembrane regions, including a large cytoplasmic loop responsible for glycosyltransferase activity and uridine 5′-diphosphate (UDP) binding [21]. HAS uses UDP-D-glucuronic acid (GlcA) and UDP-*N*-acetyl-D-glucosamine (GlcNac) to synthesize an HA chain, composed of repeating GlcA-GlcNAc units, directly into the extracellular space [21]. While all three HASs utilize this method to produce HA, they have varying enzymatic activities and produce HA of varying sizes [21,22]. HAS2 produces the largest chains compared to HAS1 and HAS3, with a molecular mass greater than 2 × 10^6^ Da [22]. HAS2 is ubiquitous throughout the body and is the most abundant HAS in the cortex [6,14,23]. HAS2 knockout is embryonic lethal due to its roles in cardiac development [14]; thus, conditional knockouts (cKO) are used to evaluate HAS2 in the brain. Brain HAS2 cKO reduces cortical HA levels and results in epileptic seizures [6,23]. In our current study, we sought to elucidate whether HAS2 synthesizes HA at newly formed synapses of developing neural circuits.

In addition to HA synthesis, it is important to understand how HA-mediated signaling regulates synaptic physiology. While HA is a space filling molecule, HA is also known to interact with cell surface receptors to mediate intracellular signaling events. The dominant cell surface receptor for HA is CD44 [24]. CD44-HA interactions regulate diverse cellular functions, including cell migration, proliferation and differentiation [25,26,27,28,29,30,31]. In non-neuronal systems, HA binding to CD44 also promotes retention of HA extracellular matrix [32]. CD44 is also known to localize to hippocampal synapses, although it is unclear whether the effects of CD44 on hippocampal synaptic plasticity are HA-dependent [31]. HA binds to *N*-terminal motifs of CD44 that act as loading sites, giving rise to clusters as it binds to HA outside the cell [33]. This alone is hypothesized to cause cellular response through changes in force on the cell surface that are balanced by intracellular attachment of CD44 to the actin cytoskeleton through ERM complexes [34]. Through these interactions, CD44 regulates RhoGTPase activity leading to actin cytoskeletal rearrangements that alter the morphology of hippocampal dendritic spines, astrocytes, breast cancer cells and keratinocytes [25,27,31,35]. Our current study demonstrates that CD44 is necessary for HA retention in developing neural circuits, where it localizes to newly formed excitatory synapses. Furthermore, similar to HAS2 and HA, CD44 suppresses excitatory synaptogenesis by attenuating the activity of the RhoGTPase, Rac1. We have previously demonstrated that elevated Rac1 activity is associated with increased excitatory synapse formation [10]. Our data establishes HA-CD44 interactions as regulators of synaptogenesis in developing neural networks. These mechanistic insights shed light on how synaptic defects may arise in disorders associated with reduced hyaluronan, leading to epilepsy, and may help to explain synaptic reductions associated with elevated hyaluronan in Alzheimer’s Disease [36,37].

## 2. Materials and Methods

### 2.1. Animals

All procedures using animals adhered to guidelines outlined in the National Research Council Guide for the Care and Use of Laboratory Animals and were approved by the Animal Care and Use Committee at East Carolina University (approval A198a) for the laboratory of Dr. Chris Geyer, from whom the samples were ob. Outbred CD-1 mice (Charles River Laboratories) were used for all studies, and the day of birth was designated as postnatal day (P)0. Mice were humanely euthanized by asphyxiation in CO2 followed by cervical dislocation. Brains from four mice at each P0 and P40 were used for imaging, both males and females were used at P0, but only males were examined at P40.

### 2.2. Cell Lines

Neurotypic control skin fibroblasts of the cell line 7545 19B were reprogrammed into human-induced pluripotent stem cells (hIPSCs) in the laboratory of Dr. Mike McConnell (University of Virginia) with the addition of Yamanaka transcription factors Oct3/4, Sox2, Klf4, and c-Myc using the CytoTune-iPS 2.0 Sendai Reprogramming Kit (Invitrogen, Waltham, MA, USA). 7545 fibroblasts were obtained under an MTA with the Coriell Institute (Camden, NJ, USA). Control WTC-11-ActBmeGFP and WTC-11-Paxilin-GFP IPSCs were obtained under MTA from the Coriell institute. The parental WTC-11 IPSC line was developed by Bruce Conklin of the Gladstone Institute and was further gene edited by the Allen Institute for Cell Science using CRISPR/Cas9 to tag endogenous β-actin or paxillin with monomeric green fluorescent protein (GFP). hIPSCs were maintained in Essential 8 Medium + E8 supplement (Gibco, Waltham, MA, USA) on hESC Matrigel (Corning, Glendale, AZ, USA) coated plates. Upon splitting, 10 μM of the ROCK inhibitor, Y27632 (Selleck Chemicals, Houston, TX, USA), was added to the cell medium.

Paxillin-GFP IPSCs were used to generate CRISPRi competent cortical spheroids via transduction of lentiviral nuclease-dead Cas9 carrying a C-terminal Krüppel associated box (KRAB) domain. (Applied Biological Materials, Richmond, BC, Canada). Expression of dCas9-KRAB transduced IPSCs was maintained by puromycin selection. Cortical spheroids were produced under puromycin selection resulting in Paxillin-GFP-dCas9-KRAB cortical spheroids. At day 90, spheroids were transduced for 48 h with lentiviral sgRNAs towards CD44 (sgCD44) to selectively repress CD44 or scrambled sgRNAs (sgScrambled) as a control at an MOI of 2.

### 2.3. Three-Dimensional Cortical Spheroid Culture

Cortical spheroids were produced following the methods described by Pasca et al. [38]. Briefly, enzymatically lifted hIPSCs were transferred to ultra-low attachment plates and cultured in DMEM supplemented with Knockout Serum Replacement (Gibco) supplemented with 5 μM Dorsomorphin (BioVision, Milpitas, CA, USA), 10 μM SB431542 (Miltenyi Biotec, Bergisch Gladbach, Germany), 10 μM Y27632 (Selleck Chemicals) for 6 days. The resulting spheroids were then maintained in neurobasal media until day 90: Neurobasal A medium, 2% B-27 supplement without vitamin A, GlutaMAX L-glutamine supplement (Gibco) penicillin/streptomycin (Gibco). Spheroids were next supplemented with 20 ng/mL of bFGF and EGF (PeproTech, Cranbury, NJ, USA) from day 6 to 25, and 20 ng/mL of BDNF and NT3 (Shenandoah Biotechnology, Warminster, PA, USA) from day 26 to 42. Spheroids were harvested beginning at day 90 for analysis.

### 2.4. HAS2 and CD44 Manipulation

Adenoviral constructs were used for the overexpression of HAS2 and CD44 in cortical spheroids. The constructs used were prepared in the same manner as Ishizuka et al., 2019 [39]. The human HAS2 ORF (NM_005328) in pCR3.1 (a gift from Dr. Tim Bowen, Cardiff University School of Medicine, Cardiff, UK) was PCR-amplified using AccuPrime Pfx DNA Polymerase (Life Technologies, Waltham, MA, USA) and primers designed specifically for use with the Adeno-X Adenoviral System 3 (Clontech, San Jose, CA, USA). The amplified human HAS2 sequence was ligated into the pAdenoX-CMV-ZsGreen1 linearized vector (Clontech) to form Ad-ZsGreen-hHAS2 and Ad-ZsGreen-hCD44. All PCR- amplified regions were verified by DNA sequencing. In the adeno-ZsGreen-HAS2 and adeno-ZsGreen-CD44 vectors, HAS2 or CD44 and ZsGreen1 sequence are present on the same plasmid but driven by separate CMV-1E promoters. Primers and plasmid for LacZ, included in the Adeno-X Adenoviral System 3 kit (Clontech) were used according to the manufacturer’s protocol to generate Ad-ZsGreen-LacZ. Cultures were transduced at 10 IFU/cell for 48 h before media washout and/or subsequent harvesting.

Knockdown of CD44 was accomplished using sgCD44 lentiviral constructs with the KRAB competent Pax-GFP cell line derived cortical spheroids. Spheroids were transduced for 48 h with pools of 3 target sgRNAs toward CD44 or Scrambled controls (Applied Biological Materials) before harvesting.

### 2.5. Immunohistochemistry

Cortical spheroids and mouse brain samples were fixed in 4% paraformaldehyde for 24 h and placed in 30% sucrose for 24 h. Samples were then embedded in OCT mounting media overnight (Sakura Finetek USA, Torrance, CA, USA), flash frozen, and cryosectioned into 10 μm thick sections. Cryosections were permeabilized with 0.2% TritonX-100 in 1× PBS before immunostaining. Primary antibodies were diluted in 2% normal goat serum in PBS, added to fixed cultures and kept at 4 °C overnight. After three PBS washes, secondary antibodies diluted in 2% normal goat serum in PBS were added to fixed cultures and kept at room temperature for 1 h. Sections fated for dSTORM imaging were additionally post-fixed in 4% paraformaldehyde and 0.1% glutaraldehyde in PBS for 10 min and subsequently washed 3 times in PBS. Cryosections were mounted using Fluoro-gel II with DAPI mounting medium (Electron Microscopy Sciences, Hatfield, PA, USA) for confocal imaging or Vectashield without DAPI (Vector Laboratories, Burlingame, CA, USA, 101098-042) for dSTORM imaging.

Two-dimensional cultures were fixed after 1 week in 4% paraformaldehyde and 4% sucrose in PBS. Cells were permeabilized with 0.2% TritonX-100 in PBS before staining. Primary antibodies were diluted in 2% normal goat serum in PBS, added to fixed cultures and kept at 4 °C overnight. After three PBS washes, secondary antibodies diluted in 2% normal goat serum in PBS were added to fixed cultures and kept at room temperature for 1 h. Cultures were mounted using Fluoro-gel II with DAPI mounting medium (Electron Microscopy Sciences).

HA was visualized using Hyaluronic Acid Binding Protein (HABP). HABP is a biotinylated link protein G1 domain of the proteoglycan versican, which binds selectively to HA. Fluorescently labeled streptavidin was used to detect HABP. See antibody table for further information. After immunostaining, cryosections were subsequently imaged using a ZEISS LSM 700 or LSM 800 confocal or Nikon Ti2-E inverted dSTORM microscope. All immunohistochemistry was performed in triplicate on at least three different sets of spheroids.

### 2.6. Confocal Microscopy

Cortical spheroid sections were imaged on a ZEISS LSM 700 confocal microscope at 40× total magnification, using the 639, 555, 488 and 405 channels. Z-stacked images were acquired, 5 z-planes with a 1 μm step size, and merged in ImageJ to generate maximum intensity projections. Confocal images were further analyzed using Image J analysis software. Each 4-channel image was analyzed as an 8-bit tiff-file. All samples of the same experiment had equal threshold values for each channel. Ten area samples measuring 100 μm in diameter were used to determine the intensity and density of the fluorescent markers along the edge of the cortical spheroid, coinciding with the cortical plate location. Spheroids were stained with pre- and post-synaptic markers to analyze the total synapse area we used the ImageJ colocalization plug in. This produced an image indicating areas at the threshold of pre- and post-synaptic markers colocalized by at least 10% of their intensities (shown in yellow in figures). The area of each individual marker as well as the colocalized synapse area was measured and normalized to DAPI. Pixel resolution for all confocal images is 0.1563257 μm per pixel.

### 2.7. Airyscan Microscopy

Three-dimensional reconstruction of HA at excitatory synapses was performed using a ZEISS LSM 800 confocal with Airyscan feature at 63× total magnification using the 639, 555 and 488 channels. Z-stacked images were acquired. Airyscan processed images were then analyzed using the 3D visualization plug-in. Pixel resolution for all Airyscan images is 0.043 μm per pixel.

### 2.8. Three-Dimensional dSTORM Microscopy

As we have previously described [9,10,11], 3D direct STochastic Optical Reconstruction Microscopy (dSTORM) images of 10 μm thick cortical spheroid sections were acquired with a Nikon Ti2-E inverted microscope with an L-APPS H-TIRF attachment and 4-line (405, 488, 561, and 640 nm) LUN-F laser module and 100 × 1.49NA Apo TIRF objective. The raw images are collected with the 3D STORM lens to a back-thinned Princeton Instruments Pro- EM-HS EMCCD 512 × 512 camera and acquired and analyzed with NIS-Elements STORM modules. The following secondary fluorophores were used for dSTORM: Atto488, Alexa568, and Alexa647, and the mounting media vectashield was used for its ability to prolong blinking [40,41,42]. The acquired images were visually inspected for spectral separation that could interfere with analysis of synaptic localization. Processed .nd2 files were analyzed in ImageJ. The .nd2 files were opened as a hyperstack, and a 3D max intensity projection was then used to plot the brightest point with the *Y*-axis as the axis of rotation. A Gaussian blur filter with a radius of 2.00 pixels was applied to avoid biasing our analysis toward a single bright link. The plot profile function of ImageJ was used to analyze the intensity of synaptic molecules perpendicular to the synaptic cleft. Average pixel resolution for exported dSTORM images of individual synapses prior to image analysis is ~0.002 μm per pixel.

For analysis of dSTORM HA localization, the following classification was used: HA whose centroid distance to that of each synaptic marker was less than 50 nm is referred to as equidistantly located in the synaptic cleft. HA whose centroid distance to that of each synaptic marker greater than 50 nm and less than 100 nm is referred to as equidistantly located near the synaptic cleft. Finally, HA centroid distance difference greater than 100 nm is referred to as preferentially located to either the pre-synaptic or post-synaptic compartment (Supplementary Figure S1).

### 2.9. Microelectrode Array Analysis

For MEA at analysis, spheroids at day 76 were dissociated using a Primary Neuron Isolation Kit (Pierce Thermo Scientific, Waltham, MA, USA, MAN0016221) and plated onto PEI-coated MEA plates prepared as described below. An Axion BioSystems Maestro Edge Multielectrode Array System (MEA) was used to record spontaneous action potentials in dissociated spheroids. MEA plates were prepared 48 h before cells were plated as follows: Wells were incubated 1 h at 37 °C with 0.1% polyethylenimine in ddH2O and rinsed 4 times with sterile water and left at room temperature overnight. After 24 h, 5 μg/mL laminin was added to wells and left overnight at room temperature. Wells were washed twice with sterile PBS before 250,000 cells/well of dissociated spheroids were added. Cultures were given MEA medium consisting of Neurobasal A medium, 2% B-27 Plus supplement without vitamin A, glutaMAX L-glutamine supplement (Gibco), and penicillin/streptomycin (Gibco). Cells were acclimated to the plates for 2 weeks before treatment and recording. Manipulation of HA levels was conducted for 24 h. Wells were recorded 10 min/h for 24 h. After treatments wells were given fresh media. Washout was recorded for 24 h. (10 min/h). Wells were treated with 4 μM TTX and immediately recorded for 10 min to suppress action potential formation and verify that the recorded electrical currents correspond to spontaneous neural activity. MEA experiments were performed on 6 sets of differentiated spheroids.

### 2.10. Western Blot Analysis

Total protein was extracted using cell lysis buffer containing protease and phosphatase inhibitor mixtures. Equivalent protein concentrations were loaded into 4–12% NuPAGE Novex Tris acetate gradient gels (ThermoFisher Scientific, Waltham, MA, USA) for electrophoresis. Proteins were subsequently transferred to a nitrocellulose membrane using a Criterion blotter apparatus (Bio-Rad, Hercules, CA, USA). The membrane was incubated in 5% non-fat dry milk in TBST for 1 h. Immunoblots were incubated overnight with primary antibody in TBST at 4 °C. The next day immunoblots receive 3–5 min washes in TBST before incubation with secondary antibody diluted in TBST for 1 h. Detection of immunoreactive bands was performed using chemiluminescence (Novex ECL, Invitrogen, Waltham, MA, USA). Blots were stripped using Restore Plus Western Stripping Buffer (Thermo Fisher Scientific) for 5–10 min at room temperature and reprobed using another primary antibody. Developed X-ray films were imaged and digitized using a Bio-Rad GelDoc with ImageLab software. Pixel intensities for bands were used for quantification after normalization to loading control bands (α-tubulin). Active Rac1-Pulldown was performed as per manufacturer instructions using Rac1 Pull-Down Activation Assay Biochem Kit (Cytoskeleton Inc., Denver, CO, USA).

### 2.11. Quantitative Real Time PCR Analysis RT-PCR (qRT-PCR)

Total RNA was isolated from cortical spheroid or 2D cultures using a Direct-zol RNA Isolation Kit (Zymo Research, Irvine, CA, USA) according to the manufacturer’s instructions. Total RNA was reverse-transcribed to cDNA using the iScript cDNA synthesis kit (Bio-Rad). Quantitative PCR was performed using Sso-Advanced SYBR-Green Supermix (Bio-Rad) and amplified on a StepOnePlus real-time PCR system (Applied Biosystems, Waltham, MA, USA) to obtain cycle threshold (Ct) values for target and internal reference cDNA levels. See table for primer sequences.

### 2.12. Statistical Analyses

Statistical analyses were performed using SigmaPlot 13.0 Software. Data sets were first tested for normality using Shapiro–Wilk tests. T-tests were run on parametric data to determine statistical significance. Non-parametric data was analyzed using Mann–Whitney Rank Sum tests to test for statistical significance.

## 3. Results

### 3.1. HA Localizes to the Synaptic Cleft of Excitatory Synapses during Cortical Development

**Figure 1:**
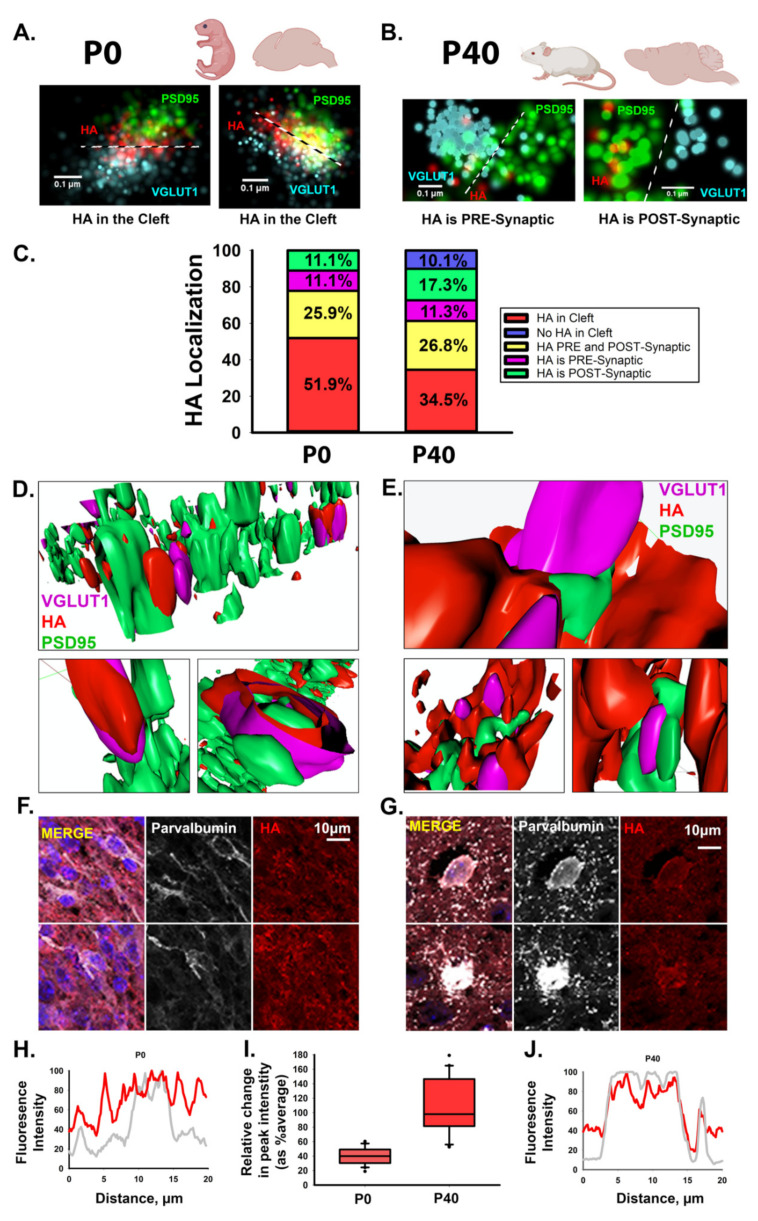
Hyaluronan exhibits a dynamic developmental expression at excitatory synapses. (**A**) Representative dSTORM images of HA within the synaptic cleft between excitatory synaptic markers, VGLUT1 and PSD95, in mouse P0 cortical slices. Cyan: pre-synaptic marker VGLUT1; green: post-synaptic marker PSD95; red: HA. (**B**) Representative dSTORM images of HA outside of the synaptic cleft, yet near synapses, in P40 mouse cortical slices. (**C**) To illustrate the shift in HA localization from P0 to P40 we used percent stacked bar charts to look at the distribution of HA localization at excitatory synapses visualized by dSTORM microscopy. Percentages are based on the distance from HA to each synaptic marker. HA in the cleft between pre- and post- synaptic is shown in red. HA near (within 100 nm) of the pre- and post-synaptic marker is labeled as HA localized to both the pre- and post-synaptic compartment (yellow). HA preferentially localizing to one synaptic compartment is labeled as HA localized to either the pre- or post- synaptic compartment (presynaptic: magenta; postsynaptic: green). Synapses lacking HA are labeled in blue. *n* = 168 synapses from 4 brain samples for each P0 or P40 time point. (**D**,**E**). Three-dimensional reconstruction of mouse excitatory synapses using Airyscan processing reveals that HA is clearly present between synaptic markers on postnatal day 1 (P0) (**D**) but shifts to surround excitatory synapses at P40 (**E**). (**F**–**J**) Inhibitory interneurons in P0 (**F**) and P40 (**G**) mouse cortical brain slices, stained for DAPI in blue, parvalbumin (inhibitory interneurons) in white, and HA in red. While HA is ubiquitous around the cells at P0, condensed perineuronal nets are absent. By contrast, HA is found in the form of a visibly condensed perineuronal net around the soma at P40.

Our previous work demonstrated that HA localizes to the synaptic cleft of newly formed excitatory synapses in human cortical spheroids that resemble the fetal brain [11]. Given that HA was previously thought to be absent from synapses [14,15], we sought to further clarify the synaptic localization of HA during cortical brain development. Notably, previous studies relied on electron micrographs of isolated adult synaptosomes or isolated synaptic junctions [15,43]. Given the hydrophilic nature of HA, dehydration techniques for EM make it difficult to preserve HA-based structures [44]. However, with the advent of super-resolution fluorescence microscopy techniques, we can now visualize HA without tissue dehydration [11]. We therefore employed both dSTORM super-resolution microscopy and Airyscan processing to visualize HA at developing synapses of the neonatal (P0) mouse cortex and mature synapses in the young adult (P40) mouse cortex (Figure 1). Consistent with our previous studies in human cortical spheroids, we used PSD95 to visualize the post-synaptic compartment and VGLUT1 to visualize the pre-synaptic compartment of excitatory synapses (Figure 1A–E). As with nascent excitatory synapses in the human cortical spheroids, hyaluronan is present in the synaptic cleft of immature excitatory synapses of the neonatal mouse cortex (Figure 1A). However, in the adult mouse brain, we observed decreased HA within the synaptic cleft, corresponding with either no synaptic HA or a redistribution to regions surrounding the synapse (Figure 1B), as has been previously described for peri-synaptic extracellular matrix [16,17]. In certain cases, HA was completely absent from excitatory synapses of the young adult mouse cortex. We quantified the frequency of synaptic localization events in P0 and P40 excitatory synapses. At immature synapses of the neonatal cortex, HA was present at all synapses examined (Figure 1C). In the majority (~80%) of cases, HA was at (51.9%) or near (25.9%) the synaptic cleft. By contrast, HA was only found in the synaptic cleft of approximately one third of mature synapses of the young adult cortex, and there was a ~20% overall reduction in HA at or near the synaptic cleft. Furthermore, HA was completely absent from ~10% of all adult excitatory synapses. To further elucidate this developmental shift from HA cleft localization to peri-synaptic matrix, we used Airyscan imaging and 3D reconstruction to visualize a larger synaptic population (Figure 1D,E). These images corroborate our dSTORM findings, with HA at or near the cleft of most immature synapses but later encapsulating mature synapses. Consistent with these synaptic changes, we also observed the emergence of a condensed hyaluronan matrix in perineuronal nets surrounding parvalbumin interneurons in the young adult mouse cortex (Figure 1F–J). Thus, hyaluronan exhibits a developmentally regulated localization at excitatory synapses, with HA predominantly found within the synaptic cleft of immature synapses but this localization to the synaptic cleft is reduced in mature synapses of the adult brain. Having established that HA exhibits a conserved and developmentally regulated localization to excitatory synapses, we sought to address how HA is synthesized at excitatory synapses, and the mechanisms through which it elicits synaptic signaling events.

### 3.2. Manipulation of Hyaluronan Synthase 2 Alters HA Synthesis, Synapse Formation, and Excitability of Developing Neural Networks

**Figure 2:**
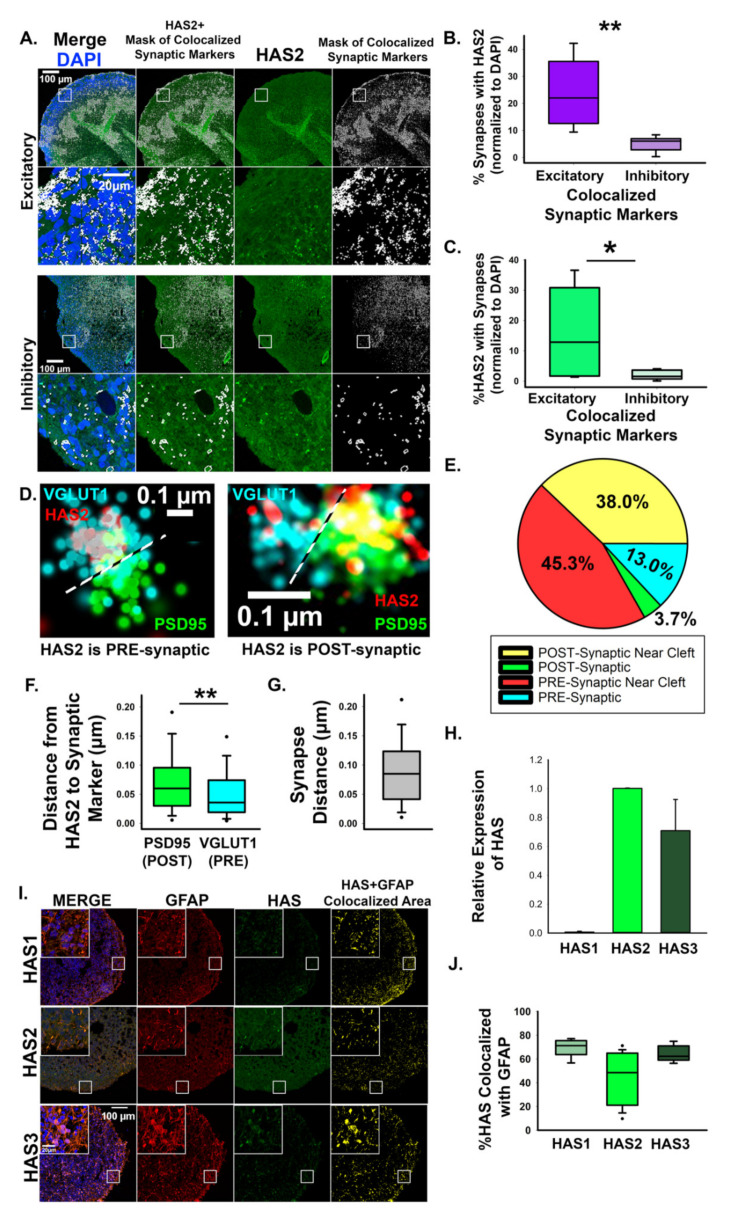
Hyaluronan synthase, HAS2, localizes to excitatory synapses. (**A**) Representative confocal images of HAS2 together with colocalized excitatory synapse markers, VGLUT1 and PSD-95, and colocalized inhibitory synapse markers, VGAT and gephyrin. Panels show DAPI (blue), HAS2 (green) and a mask of colocalized synaptic markers (white). Co-localization analysis between HAS2 and the area defined by the colocalized synapse mask reveals that HA is preferentially enriched at excitatory synapses. (**B**,**C**) Quantification of HAS2 synaptic localization shown in A. *n* = 9 total spheroids derived from 3 separately grown spheroid sets for each set of synaptic markers. * *p* = 0.027 ** *p* < 0.001. (**D**) dSTORM imaging reveals that HAS2 localizes to pre- and post-synaptic compartments of excitatory synapses. The pre-synaptic marker VGLUT1 is shown in blue, HAS2 is shown in red, and post-synaptic PSD95 is shown in green. (**E**) Pie chart showing preferred synaptic localization of HAS2. Within 100 nm of the synaptic cleft is distinguished as ‘near cleft’. (**F**) Quantification of the average distance between HAS2 and synaptic markers. *n* = 168 synapses from 9 total spheroids derived from 3 separately grown spheroid sets, ** *p* < 0.001. (**G**) Quantification of the average distance from pre- (VGLUT1) to post-synaptic (PSD95) markers demonstrates that HAS2 is closer to the cleft at the pre-synaptic VGLUT1-postive compartments. (**H**) qRT-PCR relative mRNA expression of HAS1 and HAS3 as compared to HAS2. (**I**) Representative images of cortical spheroids stained for HAS isoforms (green), GFAP (red), and DAPI. Far right panel (yellow) is the area of colocalization between HAS and GFAP. Scale bar 100 µm, inset: 20 µm. (**J**) Quantification of HAS and GFAP colocalization.

We previously established that 3D cortical spheroids generate an endogenous HA-based extracellular matrix (ECM) and that they express HAS2, the hyaluronan synthase primarily responsible for cortical HA production [6]. Using monolayer cultures, we demonstrated that HAS2 is expressed by human cortical neurons [11], consistent with research demonstrating that primary rodent cortical neurons express HAS 1–3 [45]. Using confocal image analysis, we observed significant association of HAS2 with excitatory synapses expressing VGLUT1 and PSD95 (Figure 2A–C), while only ~5% of inhibitory synapses exhibited HAS2 expression (Figure 2B). To confirm that HAS2 is directly at excitatory synapses, we used dSTORM super-resolution microscopy to visualize excitatory synapses (Figure 2D–G). We immunostained for PSD95 to visualize the post- synaptic compartment and VGLUT1 to visualize the pre-synaptic compartment of excitatory synapses. dSTORM image analysis reveals that HAS2 directly associates with the synaptic compartment of neurons and is preferentially localized to the pre-synaptic compartment in ~58.3% of HAS2-expressing synapses. To investigate contributions of other HAS isoforms, HAS1 and HAS3, we compared relative mRNA expression of HAS1 and HAS3 to levels of HAS2 (Figure 2H). HAS1 was present at low levels compared to HAS2 expression. HAS3, while more abundant than HAS1, is expressed at lower levels compared to HAS2. To determine if the HAS isoforms were associated with astrocytes rather than neurons, we stained cortical spheroids for GFAP, DAPI and each HAS isoform (Figure 2I). We found that nearly 80% of HAS1 and HAS3 expression is colocalized with astrocyte marker GFAP (Figure 2J), while HAS2 astrocytic association tends to be less. While we cannot exclude the possibility that HAS3 also regulates synaptic HA levels, these data suggest that HAS2 is the predominant HAS in cortical spheroids, where we have found it localized to both astrocytes and neurons. Using dSTORM, we have also now demonstrated that HAS2 localizes directly to excitatory synapses.

**Figure 3:**
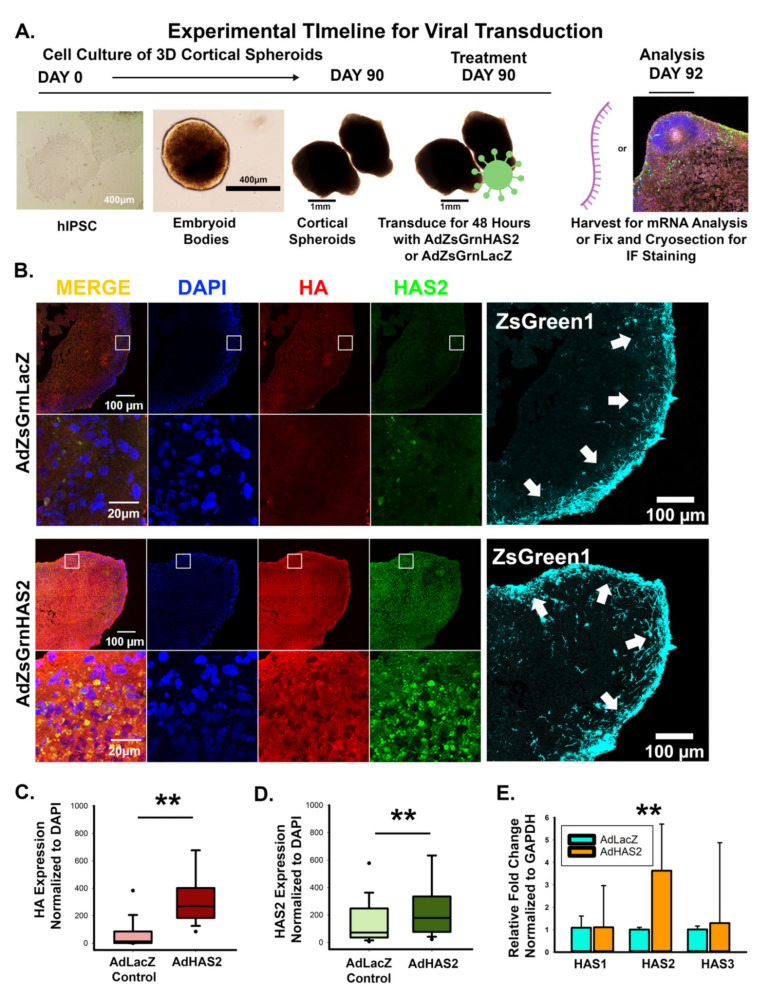
(**A**) Workflow for viral transduction of 3D cortical spheroids for manipulation of HAS2. (**B**) Representative images of cortical spheroids virally transduced to express HAS2. Top panel: adenoviral expression of ZsGreen and LacZ control (AdZsGreenLacZ). Bottom panel: adenoviral expression of ZsGreen and HAS2 (AdZsGreenHAS2), stained for DAPI (blue), HA (red), and HAS2 (green). The highlighted region of the cortical plate is enlarged below. To the right is the viral co- expression of the fluorophore, ZsGreen1. Arrows indicate the cortical plate, where synaptic analysis was performed. Scale bars top: 100 μm, enlarged region: 20 μm. (**C**) Quantified HA expression (via HABP) normalized to DAPI. *n* = 9 total spheroids for each AdZsGrnLacZ and AdZsGrnHAS2 derived from 3 separately grown and transduced spheroid sets ** *p* < 0.001. (**D**) Quantification of HAS2 expression normalized to DAPI. *n* = 9 total spheroids for each transduction (AdZsGrnLacZ and AdZsGrnHAS2). Spheroids were derived from 3 separately grown and transduced spheroid sets ** *p* < 0.001. (**E**) qRT-PCR confirmation of increased HAS2 expression. *n* = 20 total spheroids derived from 4 separately grown and transduced spheroid sets. ** *p* < 0.001.

Having established that HAS2 localizes to excitatory synapses of developing neural circuits, we sought to determine whether HAS2 is sufficient to increase hyaluronan production and alter synaptogenesis. We used adenoviral constructs to successfully transduce cortical spheroids and drive the expression of HAS2 or a LacZ control (Figure 3). In order to alter HAS2 expression during synaptogenesis, we transduced cortical spheroids at Day 90, when we observe robust synapse formation that it is susceptible to exogenous manipulations [9,10,11] (see Figure 3A for detailed workflow). RT-PCR confirmed that *HAS2* expression significantly increased 48 h after transduction (Figure 3E). Notably, *HAS1* and *HAS3* were unaltered by exogenous *HAS2* transduction. To visualize the location of increased HAS2 expression, we imaged the adenoviral expression of the virally co-expressed fluorophore, ZsGreen. ZsGreen expression is prominent in outer cortical layers, where synaptogenesis occurs in our model [10,11,12] (arrows in Figure 3B). Through immunostaining, we confirmed that HAS2 protein expression increased and resulted in increased hyaluronan production in the cortical plate where synaptogenesis occurs (Figure 3C,D). Increased HA levels demonstrate that the expressed HAS2 is functional.

**Figure 4:**
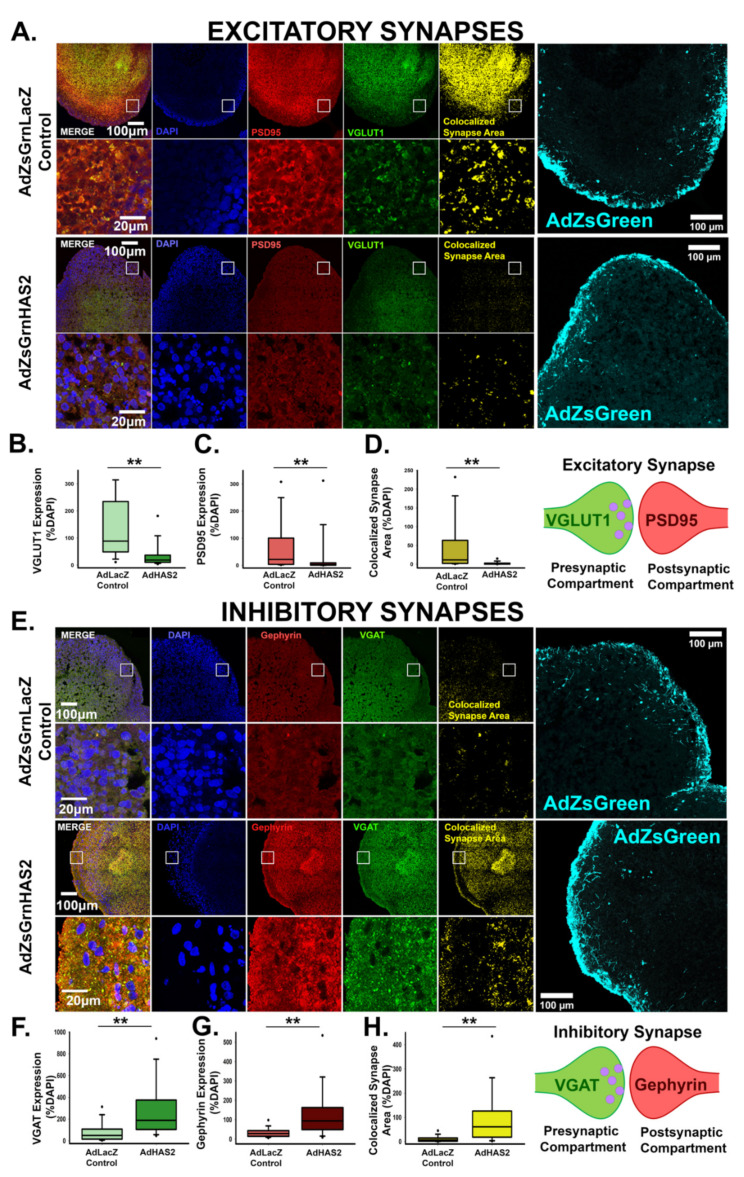
Hyaluronan synthase, HAS2, alters the emerging balance between excitatory and inhibitory synapses in developing neural networks. (**A**) Representative images of cortical spheroids transduced with either LacZ control or HAS2 and stained for DAPI (blue) and excitatory synapses: post-synaptic marker PSD95 (red) and pre-synaptic marker VGLUT1 (green). Right panel shows the excitatory synaptic colocalization of PSD95 and VGLUT1 in yellow. Bottom panel highlights the region of the cortical plate indicated by the white box. Scale bars: top: 100 μm, enlarged region: 20 μm. (**B**) Quantification of the area of pre-synaptic marker VGLUT1 normalized to the area of DAPI. (**C**) Quantification of area of post-synaptic marker PSD95 normalized to the area of DAPI. (**D**) Quantification of colocalized excitatory synapse area normalized to the area DAPI. *n* = 9 total spheroids for each transduction (AdZsGrnLacZ and AdZsGrnHAS2). Spheroids were derived from 3 separately grown and transduced spheroid sets ** *p* < 0.001 for (**B**–**D**). (**E**) Representative images of transduced cortical spheroids stained for DAPI (blue), and inhibitory synapses: post-synaptic marker gephyrin (red) and pre-synaptic maker VGAT (green). Rightmost panel shows the inhibitory synaptic colocalization of gephyrin and VGAT in yellow. Bottom panel highlights the region of the cortical plate indicated by the white box. Scale bars: top: 100 μm, enlarged region: 20 μm. (**F**) Quantification of the area of pre- synaptic marker VGAT normalized to the area of DAPI. (**G**) Quantification of the area of post- synaptic marker gephyrin normalized to DAPI. (**H**) Quantification of colocalized inhibitory synapse area normalized to the area of DAPI. *n* = 9 total spheroids for each transduction (AdZsGrnLacZ and AdZsGrnHAS2). Spheroids were derived from 3 separately grown and transduced spheroid sets ** *p* < 0.001 for (**F**–**H**).

Given that elevated levels of HA suppress excitatory synapse formation [11], we anticipated that HAS2-mediated increases in HA would similarly impact synaptogenesis. We immunostained for pre- and post-synaptic markers, VGLUT1 and PSD95, of excitatory synapses in cortical spheroid cryosections 48 h after viral transduction, when we observe both increased HAS2 and HA (Figure 3) and analyzed the colocalized area between pre- and post-synaptic markers, to determine the extent of synapse formation (Figure 4A–D). When compared to the LacZ control, HAS2 overexpression significantly decreased the area of individual pre- and post-synaptic excitatory markers as well as their colocalized synaptic area, highlighting a regulatory role for HAS2 in excitatory synapse formation. Notably, our human cortical spheroids resemble dorsal forebrain development and therefore predominantly form excitatory synapses [38,46]. However, we are still able to observe inhibitory synapses, albeit fewer than excitatory synapses [9,11]. To visualize inhibitory synapses, we immunostained for inhibitory pre- and post-synaptic markers, vesicular GABA transporter (VGAT) and gephyrin, respectively (Figure 4E–H). Notably, inhibitory synapses dramatically increase in response to HAS2 overexpression. These data demonstrate that HAS2- mediated HA synthesis at excitatory synapses can significantly alter the emerging balance between excitatory and inhibitory synapses in developing neural networks.

**Figure 5:**
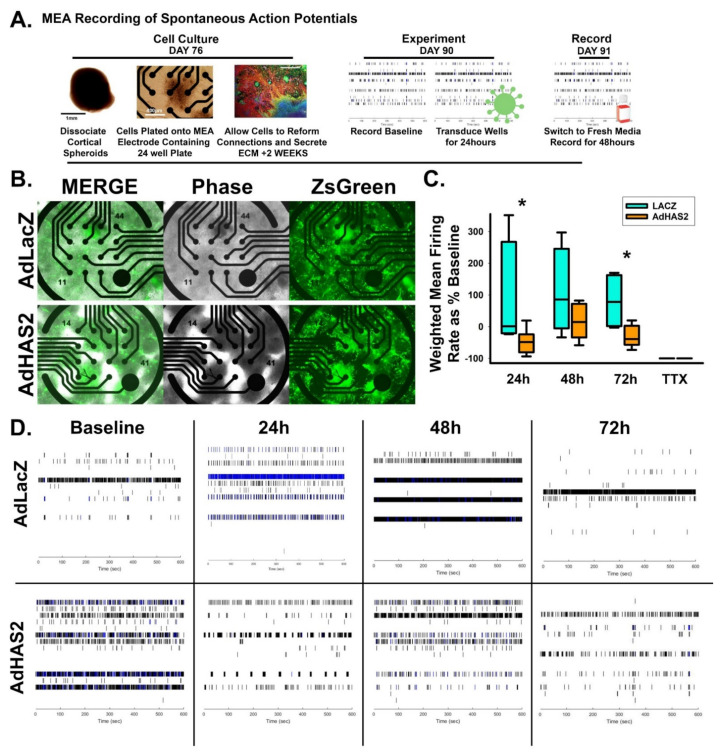
HAS2 suppresses excitability of developing neural networks. (**A**) Workflow of multielectrode array experiments for detection of action potentials propagation in neural networks. (**B**) Representative images of successful viral transduction on MEA plates as indicated by viral expression of ZsGreen in dissociated spheroid transduced with adenovirus constructs for control LacZ (AdZsGrnLacZ) and HAS (AdZsGrnHAS2) expression. (**C**) Quantification of weighted mean firing rate of spontaneous action potentials in HAS2 and LacZ-expressing dissociated spheroids demonstrates that HAS2 suppresses excitability by significantly reducing the firing rate at 24 and 72 h post-transduction. Tetrodotoxin (TTX) was used to inhibit action potential propagation, demonstrating that the firing rate reflects action potentials. *n* = 6 wells of dissociated spheroids per treatment. Spheroids were derived from 3 separately grown and transduced spheroid sets. * *p* ≤ 0.05 (**D**) Representative raster plots of spontaneous action potential firing at 0 (baseline), 24, 48, and 72 h post-transduction for AdZsGrnLacZ and AdZsGrnHAS2.

To determine whether these synaptic changes correspond with altered synaptic transmission, we used microelectrode array analysis to measure spontaneous action potential firing rate in LacZ and HAS2-transduced cultures of dissociated cortical spheroids (detailed workflow described in Figure 5A, representative image of transduced dissociated cultures shown in Figure 5B). We have previously established that dissociated cortical spheroid re-secrete and retain an HA-based extracellular matrix that regulates neuronal activity [11]. Control LacZ adenoviral transduction decreases HA levels and increases neuronal activity when compared to untreated spheroids (data not shown). Thus, we compared the effects of adenoviral expression of HAS2 (Figure 5) and later CD44 (Figure 8) with the appropriate LacZ control. Within developing neural circuits of human cortical spheroids, spontaneous action potentials form and increase with further development. We predicted that the decreased excitatory synapse formation resulting from HAS2 overexpression would prevent spontaneous action potential formation. HAS2 overexpression significantly reduced spontaneous activity, decreasing activity by ~50% at both 24 and 72 h after viral transduction (Figure 5C), as illustrated by the raster plots of neural spiking activity in Figure 5D. These results suggest that HAS2 represses the development of spontaneous activity through synaptic HA synthesis. However, whether synaptic HA functions solely as a physical barrier or also interacts with synaptic receptors to alter synapse formation remains to be elucidated.

### 3.3. HA-CD44 Interactions at Developing Excitatory Synapses Attenuate Synaptogenesis through Regulation of RhoGTPase Signaling

**Figure 6:**
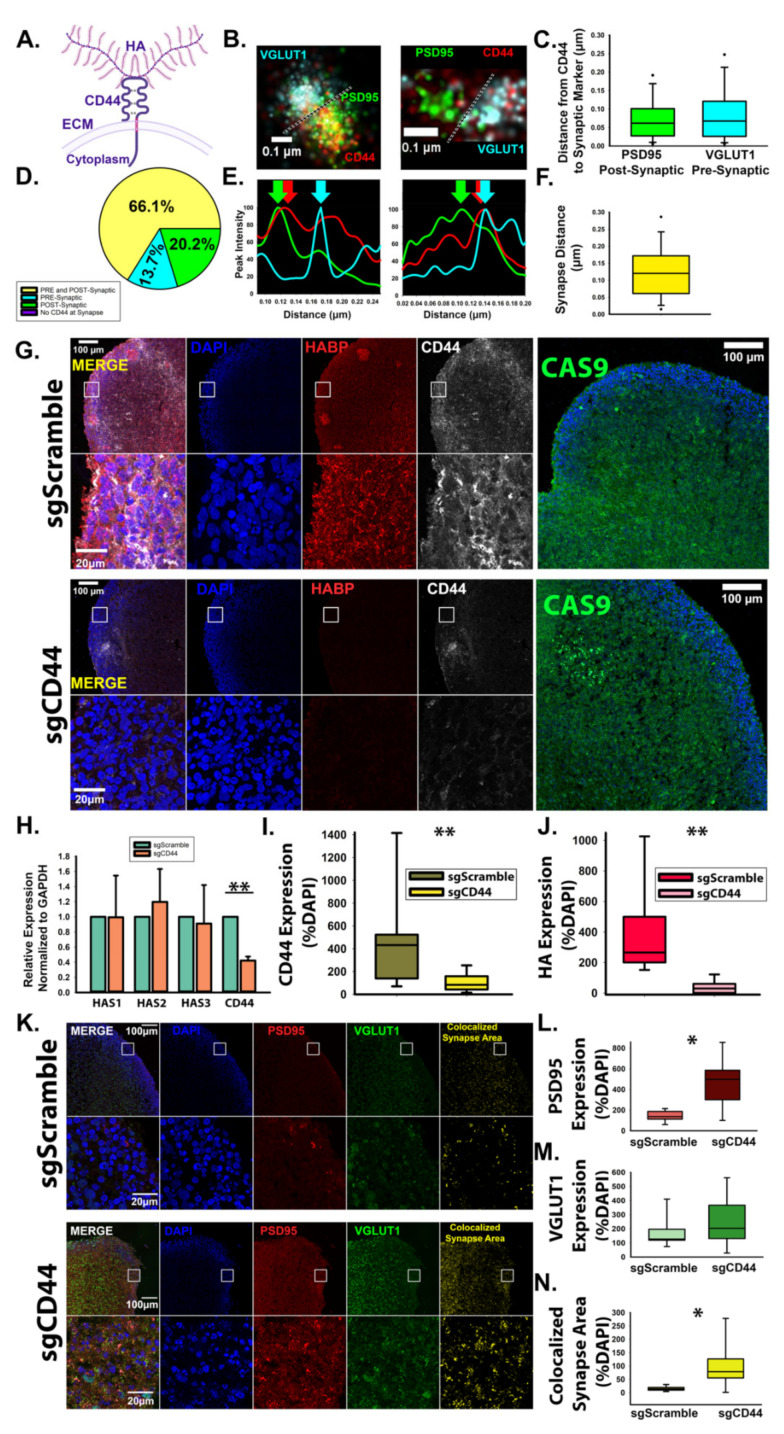
CD44 localizes to excitatory synapses and promotes HA retention in developing neural circuits. (**A**) Schematic of transmembrane protein CD44 bound to HA. (**B**) Representative dSTORM images of CD44 at excitatory synapses demonstrates that CD44 localizes to both pre- and post-synaptic compartments. Green: PSD95; red: CD44; cyan: VGLUT1. (**C**) Quantified distance from CD44 to pre- and post-synaptic markers. *n* = 168 synapses from 9 total spheroids derived from 3 separately grown spheroid sets. (**D**) Quantification of CD44 localization at individual excitatory synapses reveals that in the majority of cases, CD44 localizes to both the pre- and post-synaptic compartment (yellow) but may also be found solely within the pre- synaptic (blue) or post-synaptic (green) compartment. (**E**) Synaptic intensity plot profiles corresponding to the synapses shown in B. Arrow indicate the peak intensity of PSD95, VGLUT1, and CD44 at the synapse. (**F**) Quantification of the synaptic distance between VGLUT1 and PSD95. *n* = 168 synapses from 9 total spheroids derived from 3 separately grown and transduced spheroid sets. (**G**) Representative images of cortical spheroids stably expressing dCas9-KRAB (green) for CRISPR-mediated interference of gene expression. At day 90, dCAS9-KRAB-expressing cortical spheroids were lentivirally transduced with either non- targeting scrambled sgRNA or sgRNA directed toward the promoter region of CD44. After 48 h, spheroids were fixed and stained for DAPI (blue), HABP (red) and CD44 (white). (**H**) qRT- PCR confirmation of CD44 knockdown. *n* = 9 total spheroids for each sgScramble and sgCD44 derived from 3 separately grown and transduced spheroid sets ** ≤ 0.001. (**I**) Quantification of area of CD44 expression normalized to the area of DAPI. *n* = 18 ** *p* < 0.001. (**J**) Quantification of area of HABP expression normalized to DAPI area. *n* = 9 total spheroids for each sgScramble and sgCD44 derived from 3 separately grown and transduced spheroid sets ** *p* < 0.001. (**K**) Representative images of cortical spheroids stained for excitatory synaptic markers, PSD95 and VGLUT1, and DAPI. Far right panel: colocalized synaptic area. (**L**) Quantification of PSD95 expression normalized to DAPI. * *p* < 0.05. (**M**) Quantification of VGLUT1 expression normalized to DAPI. (**N**) Quantification of colocalized excitatory synapse area normalized to DAPI, * *p* < 0.05.

**Figure 7:**
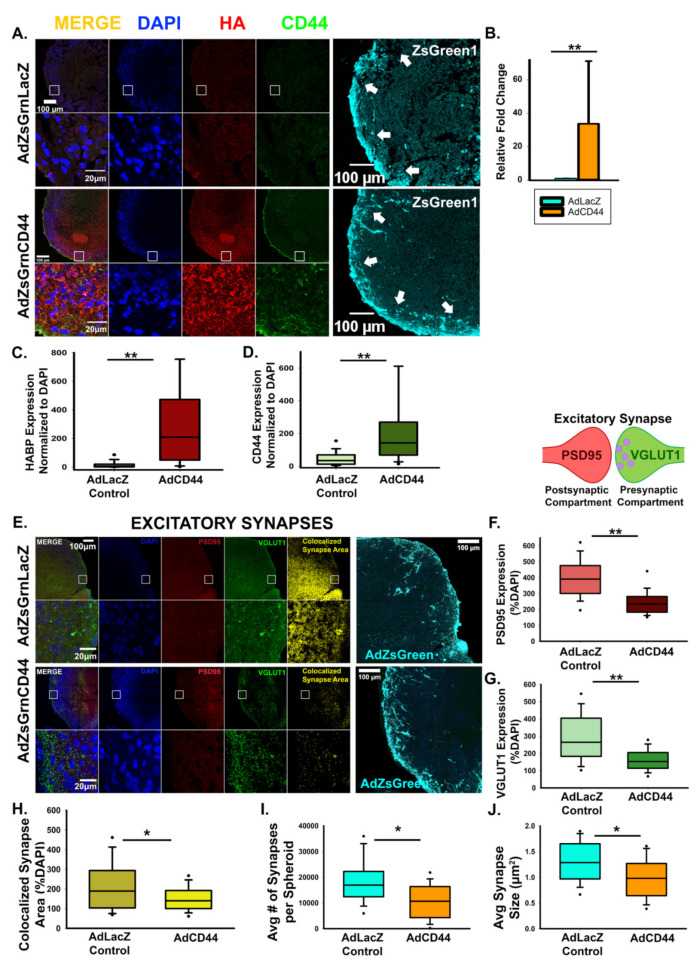
CD44 antagonizes excitatory synapse formation. (**A**) Representative images of cortical spheroids transduced with either LacZ control or CD44 and stained for DAPI (blue), HA (red), and CD44 (green). Highlighted region of the cortical plate is enlarged below. Successful transduction was confirmed via viral expression of the fluorophore, ZsGreen1 (cyan, far right panels). Arrows indicate the cortical plate, where synapse analysis was performed. Scale bars top: 100 μm, enlarged region: 20 μm. (**B**) qRT-PCR confirmation of increased CD44 expression normalized to GAPDH. (**C**) Quantification of the area of HA (HABP) normalized to the area of DAPI reveals that CD44 promotes HA retention. *n* = 9 total spheroids for each transduction (AdZsGrnLacZ and AdZsGrnCD44). Spheroids were derived from 3 separately grown and transduced spheroid sets ** *p* < 0.001. (**D**) Quantification of the area CD44 expression normalized to the area of DAPI. *n* = 9 total spheroids for each transduction (AdZsGrnLacZ and AdZsGrnCD44). Spheroids were derived from 3 separately grown and transduced spheroid sets ** *p* < 0.001. (**E**) Representative images of cortical spheroids transduced with either LacZ control or CD44 and stained for DAPI (blue), and excitatory synapses: post-synaptic marker PSD95 (red), and pre-synaptic maker VGLUT1 (green). Right panel shows the excitatory synaptic colocalization of PSD95 and VGLUT1 in yellow. Bottom panel highlights the region of the cortical plate indicated by the white box. Successful transduction was confirmed via viral co- expression of the fluorophore, ZsGreen1 (cyan, far right panels). Scale bars: top: 100 μm, enlarged region: 20 μm. (**F**–**H**) Quantification of individual pre- and post-synaptic markers and their co-localization in excitatory synapses reveals that CD44 antagonizes excitatory synapse formation. Quantification of the area of post-synaptic marker PSD95 normalized to the area of DAPI. *n* = 9 total spheroids for each transduction (AdZsGrnLacZ and AdZsGrnCD44). Spheroids were derived from 3 separately grown and transduced spheroid sets. ** *p* < 0.001 (**F**). Quantification of the area of the pre-synaptic marker VGLUT1 normalized to the area of DAPI. *n* = 9 total spheroids for each transduction (AdZsGrnLacZ and AdZsGrnCD44). Spheroids were derived from 3 separately grown and transduced spheroid sets. ** *p* < 0.001 (**G**). Quantification of co-localized excitatory synapse area normalized to the area of DAPI. *n* = 9 total spheroids for each transduction (AdZsGrnLacZ and AdZsGrnCD44). Spheroids were derived from 3 separately grown and transduced spheroid sets. * *p* = 0.030 (**H**). (**I**,**J**) Quantification of the average number of synapses per spheroid (**I**) and individual synapse size (**J**) demonstrates that CD44 promotes the refinement of excitatory synapses into discrete puncta. *n* = 9 total spheroids for each transduction (AdZsGrnLacZ and AdZsGrnCD44). Spheroids were derived from 3 separately grown and transduced spheroid sets. ** *p* < 0.001.

To determine whether HA and HAS2 suppress synaptogenesis via synaptic signaling events, we investigated whether the predominant HA receptor, CD44 (Figure 6A), localizes to developing excitatory synapses. Using dSTORM imaging analysis, we observed that CD44 associates with both VGLUT1-positive pre-synaptic and PSD95-positive post-synaptic compartments (Figure 6B–F). Notably, CD44 was found in all examined excitatory synapses. In the majority of excitatory synapses (~66%), CD44 localized to both the pre- and post-synaptic compartment, followed by ~20% solely in post-synaptic compartment and ~14% solely in the pre-synaptic compartment (Figure 6D). Since CD44 promotes HA retention in other tissue systems [32], we sought to determine whether CD44 is responsible for HA retention in developing neural circuits. In order to knockdown CD44 specifically during synaptogenesis, we used human cortical spheroids stably expressing a dead Cas9 enzyme conjugated to the transcriptional repressor, Krüppel-Associated Box (KRAB) for CRISPR interference [47,48]. After 90 days of development, we introduced targeting sgRNA towards the CD44 promoter, which downregulated CD44 (Figure 6G) as confirmed by RT-PCR (Figure 6H). Using immunofluorescence, we observed that this strategy led to global CD44 reductions in our cortical spheroid model (Figure 6G,I). Strikingly, loss of CD44 resulted in significant loss of HA (Figure 6J). Conversely, adenoviral expression of CD44 increased HA levels (Figure 7). These changes in HA levels are unlikely to be driven by increased HA synthesis as we did not observe changes in HAS2 expression resulting from knockdown of CD44 (Figure 6H). Knockdown of CD44 was sufficient to increase excitatory synaptogenesis (Figure 6K–N.) Together, these results demonstrate that CD44 is responsible for HA retention in the developing neural circuits of our model of the human fetal cortex and that loss of HA retention via loss of CD44 alters synaptic networks.

**Figure 8:**
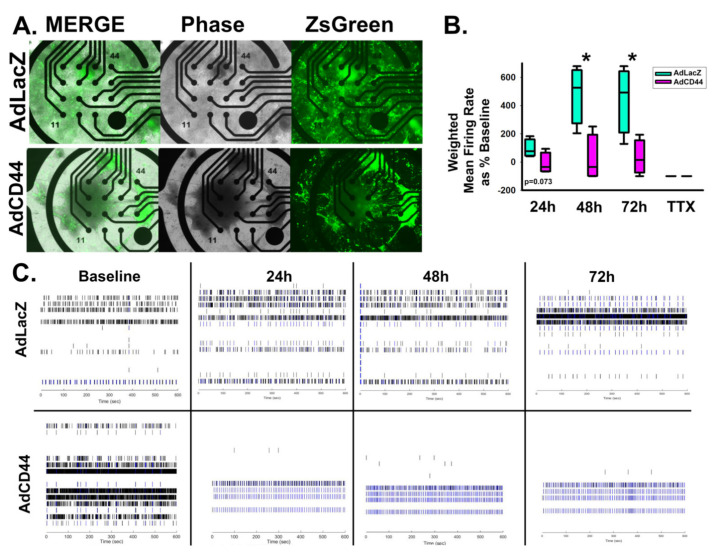
CD44 suppresses excitability of developing neural networks. (**A**) Representative images of successful viral transduction on MEA plates as indicated by viral expression of ZsGreen in dissociated spheroid transduced with adenovirus constructs for control LacZ (AdZsGrnLacZ) and CD44 (AdZsGrnCD44) expression. (**B**) Quantification of weighted mean firing rate of spontaneous action potentials in CD44 and LacZ-expressing dissociated spheroids demonstrates that CD44 suppresses excitability by significantly reducing the firing rate at 48 and 72 h post-transduction. Tetrodotoxin (TTX) was used to inhibit action potential propagation, demonstrating that the firing rate reflects action potentials. m = 6 wells of dissociated spheroids per treatment, spheroids were derived from 3 separately grown and transduced spheroid sets. * *p* < 0.05. (**C**) Representative raster plots of spontaneous action potential firing at 0 (baseline), 24, 48, and 72 h post-transduction for AdZsGrnLacZ and AdZsGrnCD44.

Notably, adenoviral CD44 expression resulted in decreased excitatory synapse formation compared to viral expression of a LacZ control. Viral transduction was confirmed via visualization of viral co-expression of ZsGreen, and the resulting CD44 overexpression was confirmed by qRT-PCR analysis (Figure 7A–D). In addition to regulating the overall area of excitatory synapses (Figure 7E–H), CD44 overexpression also decreased the size and number of individual synapses, which may suggest a role for CD44 in synaptic refinement (Figure 7I,J). Furthermore, CD44 suppressed spontaneous action potential formation similar to HAS2 and HA (Figure 8), suggesting that HAS2 and CD44 cooperatively regulate synapse formation, by HAS2-mediated HA synthesis at developing synapses followed by HA binding to CD44 for retention and potentially influencing intracellular signaling events.

**Figure 9:**
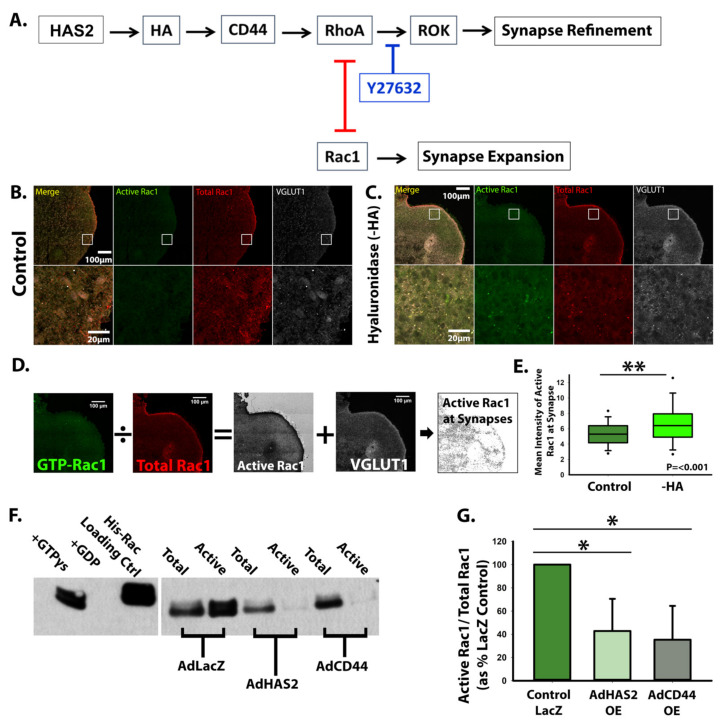
HAS2, HA, and CD44 regulate synapse formation via RhoGTPase signaling. (**A**) Graphic representation of proposed mechanism by which HAS2, HA, and CD44 regulate the antagonistic relationship between RhoGTPases, RhoA and Rac1, to promote refinement of excitatory synapses. (**B**,**C**) Control cortical spheroids (**B**) or cortical spheroids treated with streptomyces hyaluronidase (**C**) to degrade HA were immunostained for active GTP-bound Rac1 and total Rac1 together with VGLUT1 to visualize association with developing excitatory synapses. (**D**) Ratiometric imaging was performed by dividing the fluorescence intensity for GTP-Rac1 by the fluorescence intensity for total Rac1. The resulting ratiometric image intensity was measured in particles corresponding to VGLUT1 area. (**E**) Quantification of Rac1 activity associated with VGLUT1-postive compartments reveals that HA suppresses Rac1 activity, while treatment with hyaluronidase significantly increases Rac1 activity. *n* = 9 total spheroids for each treatment derived from 3 separately grown and treated spheroid sets. ** *p* < 0.001 (**F**) Immunoblot of Active Rac1 pulldown. Glutathione affinity beads were used to pulldown GTP-Rac1. Columns left to right: +GTPγs positive control, +GDP negative control, His-Rac1 antibody loading control, Total (30 μg) and Active (300 μg of lysate incubated with beads) samples for spheroids transduced with AdZsGreenLacZ, AdZsGreenHAS2, or AdZsGreenCD44. (**G**) Quantification of Active Rac1 pulldowns reveal HAS2 and CD44 overexpression decrease Rac1 activity, reported as GTPγs-Rac1/Total-Rac1 as percent of AdZsGreenLacZ control. *n* = 3 spheroids for each AdZsGrnLacZ, AdZsGrnHAS2 and AdZsGrnCD44 derived from 3 separately grown and transduced spheroid sets, ran on 3 separate western blot analyses. * *p* < 0.05.

To determine whether HA functions solely in a space-filling physical capacity or whether it can also alter intracellular signaling events, we examined RhoGTPase signaling alterations arising from HA or CD44 manipulations. CD44 is known to be tethered to the actin cytoskeleton through ERM complexes and modulate cell morphology via RhoGTPases [34,49]. We previously established that antagonistic RhoGTPase signaling regulates synaptogenesis [10]. Specifically, RhoA kinase (ROCK)-mediated actin bundling suppresses excitatory synaptogenesis, but ROCK inhibition increased Rac1 activity and excitatory synapse formation [10] (Figure 9A). Based on these data, we hypothesized that HA-CD44 interactions antagonize excitatory synapse formation by promoting RhoA activity and suppressing Rac1 activity (Figure 9A). Consistent with this hypothesis, hyaluronidase degradation of HA, which increases excitatory synapse formation, increases Rac1 activity at synapses (Figure 9B–E). Conversely, HAS2 and CD44 overexpression significantly decreased Rac1 activity (Figure 9F,G), corresponding with the observed decrease in excitatory synapse formation (Figure 4 and Figure 7). Thus, in addition to its physical properties, HA also has the ability to alter intracellular synaptic signaling events through interaction with its receptor, CD44.

## 4. Discussion

While HA is known to be substantially increased during the prenatal developmental window and to impact early events in brain development, such as neurulation [50,51,52], our research is the first to establish a role for HA in excitatory synapse formation and the emergence of synaptic activity in developing neural circuits [11]. In our current research, we sought to determine the mechanisms underlying the synthesis and signaling of HA at developing excitatory synapses. Given that HA was previously thought to be excluded from the synaptic cleft [15,43], we first demonstrate that this developmental localization is conserved between human brain spheroids and the developing mouse brain. HA localizes to the synaptic cleft of the majority of newly formed excitatory synapses in both human cortical spheroids [11] and the mouse neonatal brain (Figure 1). While human synapse formation begins prenatally during mid-fetal gestation and continues into juvenile development [12], mouse synapse formation occurs predominantly around birth [53]. Thus, we used P0 mouse cortex to capture synapse formation similar to synapse formation in our model of human mid-fetal cortical development [9,10,11,12,38]. We compared HA at excitatory synapses of the P0 mouse neonatal brain at the time of synapse formation with the young adult mouse brain (P40) during the maintenance of mature synaptic connections (Figure 1). At immature synapses of the neonate cortex, HA predominantly localizes to the synaptic cleft, corroborating our previous observations in human cortical spheroids that model fetal synaptogenesis [11]. By contrast, in the adult mouse brain, HA largely surrounds synapses, consistent with previous reports of HA in peri-synaptic extracellular matrix [16]. The peri-synaptic extracellular matrix is associated with the formation of a tripartite synapse containing an astrocytic endfoot [53]. In mice, the majority of astrocytic contributions to synaptic development occur postnatally [16,53,54]. For example, during the first and second week of postnatal development, astrocytes segregate into non-overlapping territories, with one astrocyte ensheathing up to 100,000 synapses [54,55,56]. During this time frame, there is also a robust increase in the number of synapses and a corresponding increase in synaptic activity [13,57]. Astrocytes contribute to synaptic function in part by regulating glutamate uptake from the synaptic cleft, preventing glutamate-induced neurotoxicity [58,59]. Hyaluronan supports the activity of the glial glutamate transporter [60]. Thus, while our data supports a model in which HA is directly synthesized into the developing synaptic cleft, it is interesting to speculate that with further development, HA is synthesized by associated astrocytes, which would surround and stabilize mature synaptic contacts. In addition to the developmental changes in HA synaptic localization, postnatal development also coincides with the emergence of perineuronal nets around inhibitory interneurons [14,19,61]. In early brain development, the HA-based ECM is loosely packed and forms the interstitial ECM [62] (Figure 1G) and lacks the dense meshwork of HA in perineuronal nets around the soma of interneurons (Figure 1H) [14]. Our data thus highlights a critical temporal role for HA at developing synapses, while also elucidating mechanisms of HA synthesis and signaling at these newly formed synapses. It is interesting to speculate that this early localization to synapses may be necessary for initial stages of synaptogenesis. For example, HA binding can lead to clustering of CD44 [33], and CD44 is known to interact with and regulate the expression of post-synaptic density proteins, such as PSD-95 and Bassoon [31,63]. Thus, HA-CD44 interactions may help to initiate post-synaptic density (PSD) formation. Once formed, glutamate receptors clustered within the PSD can readily respond to pre-synaptic glutamate release, leading to synaptic strengthening. Neuronal activity is associated with increased matrix remodeling, which may lead to the observed loss of HA from excitatory synapses of older animals (Figure 1) [42,64]. Future studies are needed to address whether activity impacts HA synaptic synthesis, localization and turnover. However, our current study provides a foundation for such future studies by elucidating mechanisms of HA synthesis, signaling and retention within developing neural circuits.

In cortical brain development, HAS2 is the predominant hyaluronan synthase isoform [6. Furthermore, data from the Human Brain Transcriptome demonstrates that the RNA expression of HAS2 is highest during prenatal brain development when synapses are forming (Available online: http://www.hbatlas.org, accessed on 16 September 2021) [65,66,67]. We establish that HAS2 critically regulates excitatory synapse formation and function through synthesis of HA. Using combined confocal and dSTORM microscopy, we demonstrate that HAS2 preferentially localizes to excitatory synapses, where it is found in both pre- and post-synaptic compartments (Figure 2 and Figure 3). During excitatory synapse formation, pre- and post-synaptic projections contact and adhere to one another [68]. In this developmental context, HAS2 is capable of secreting HA directly into the developing synaptic cleft. Notably, increased HA levels via introduction of high molecular weight HA [11] or overexpression of HAS2 attenuate excitatory synaptogenesis and suppress spontaneous action potential formation (Figure 4 and Figure 5). Thus, within the critical window of synapse formation in early brain development, the synaptic expression of HAS2 uniquely positions it to prevent the emergence of hyperexcitability commonly observed in neurodevelopmental disorders, such as epilepsy and autism spectrum disorders [3,4,5].

Following HA synthesis, subsequent interaction of HA with its receptor CD44 alters intracellular RhoGTPase signaling pathways that attenuate synapse formation (Figure 6 and Figure 9). dSTORM super-resolution microscopy reveals that CD44 localizes near the synaptic cleft in both the pre- and post-synaptic compartment of excitatory synapses (Figure 6), enabling both sides of the synapse to adhere to the HA-based ECM. In other cells, interactions between HA and CD44 stabilize membrane protrusions leading to directional migration [69]. Thus, we hypothesize that CD44 helps to stabilize newly formed synaptic contacts. Similar to other tissue systems [25,27,70], we also establish that CD44 is necessary for HA retention in developing neural networks (Figure 6). Together with HA retention, CD44 prevents excitatory synaptogenesis and spontaneous action potential formation similar to HAS2 and HA expression [11] (Figure 7 and Figure 8). These effects are driven by regulation of RhoGTPase signaling, as has previously been observed for CD44 in mature hippocampal neuron cultures, although it was not determined whether these effects were dependent on HA [31]. Specifically, HA and CD44 suppress Rac1 activity (Figure 9). We have previously demonstrated that Rac1 activity promotes the formation of dendritic spine precursors [7] and is associated with increased post-synaptic surface area [71] and increased synapse formation [10]. Thus, we propose that CD44 plays a critical role in synaptic refinement, stabilizing newly formed synapses but also restricting their growth and attenuating the formation of new synapses. A possible role for CD44 in synaptic refinement is supported by our observed decrease in the size of individual synapses in response to adenoviral expression of CD44 (Figure 7J). Thus, while previous HA-mediated effects on synaptic function were attributed to changes in the physical space occupied by the complex HA structure [6,23], our data also shows that HA can regulate synaptic activity through cell signaling events mediated by interaction with its receptor CD44 (Figure 9). Thus, our data highlights a critical role for synaptic HAS2-HA-CD44 in protecting developing neural networks from the emergence of hyperexcitability and epilepsy through a RhoGTPase signaling axis, by suppressing aberrant Rac1 hyperactivation [23]. These results are consistent with emerging data suggesting that RhoGTPase signaling pathways may be targeted therapeutically to restore normal synapse development and cognition in neurodevelopmental disorders [72,73]. Furthermore, these results indicate that hyaluronan pathways can be manipulated to restore synaptic function, especially in brain disorders associated with altered hyaluronan levels. For example, elevated HA or CD44-mediated retention of HA may be used to suppress elevated excitatory synaptic transmission in epilepsy [6,23]. Conversely, increased hyaluronan is observed in aging and Alzheimer’s Disease [62,74,75]. Together with this increase in hyaluronan, synapse loss is observed prior to neurodegeneration in Alzheimer’s Disease [76,77,78,79] and correlates with the severity of cognitive decline [80]. It is therefore interesting to speculate that decreased hyaluronan synthesis or CD44 may be used to prevent synapse loss.

Ultimately, our use of dSTORM super-resolution microcopy allowed us to observe a previously unappreciated localization of HA to the synaptic cleft of immature synapses in both a cell culture model of human cortical development and the mouse neonatal brain. Using our genetically tractable and physiologically relevant model of human brain development, we were able to disrupt HA synthesis and signaling specifically during synapse formation, revealing novel roles for HAS2 and CD44 in synapse formation and the emerging balance between excitatory and inhibitory signaling in developing neural circuits. Future modulation of these developing networks and their interactions with the HA-based ECM will allow us to appreciate how changes in HA synaptic localization contribute to synapse maturation and refinement.

## Data Availability

Not applicable.

## Supplementary Materials

The following are available online at https://www.mdpi.com/article/10.3390/cells10102574/s1, Figure S1: HA, Table S1: Primary Antibodies, Table S2: Secondary Antibodies, Table S3. Primer Sequences.
