# Supplementary material for: Synaptic Hyaluronan Synthesis and CD44-Mediated Signaling Coordinate Neural Circuit Development
Source: Cells. 2021 Sep 28;10(10):2574. doi: 10.3390/cells10102574 (PMC8533746; doi:10.3390/cells10102574)
Supplement: Supplementary file 1 [file cells-10-02574-s001.zip › cells-1361582-supplementary.pdf]

S. Fig. S1.

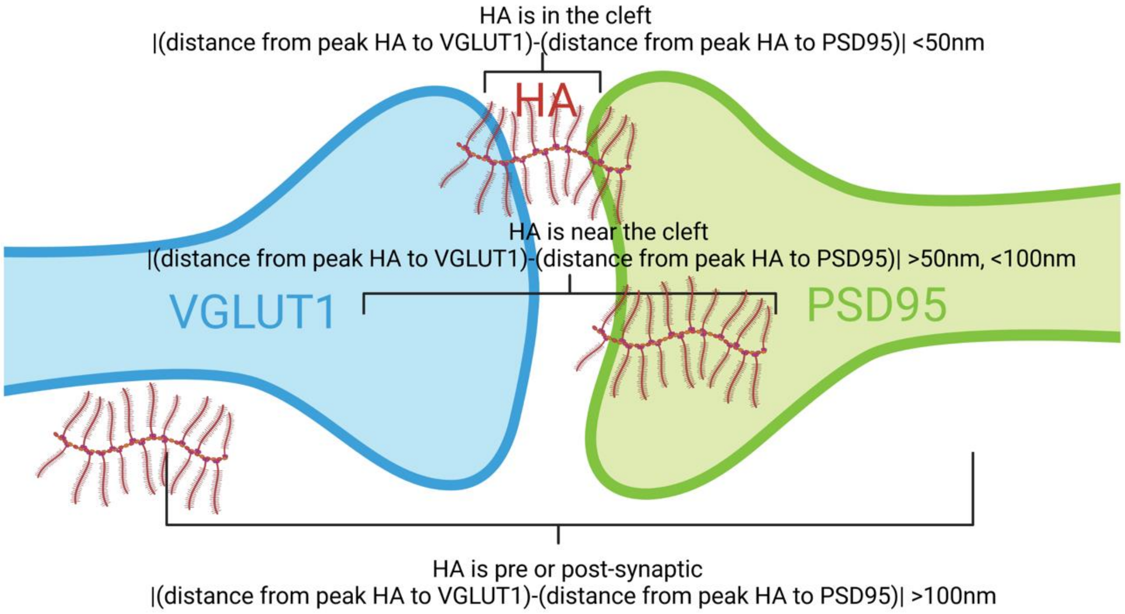

For HA that is near or definitively on one side of the synaptic cleft, the PRE or POST synaptic position is determined by the lesser value: HA distance to VGLUT1 or HA distance to PSD95.
i.e distance peakHA to peakVG= 0.002um, distance peakHA to peakPSD= 0.120um , |(0.002)-(0.120)|= 0.118. HA is presynaptic.

Supplemental Tables

S. Table S1. Primary Antibodies

Primary Antibody	Brand	Catalog Number	Host	Class	Dilution	Concentration Used
Active Rac1	NewEast Biosciences	26903 RRID:AB_1961793	Mouse IgM	Monoclonal	1:500*	1μg/mL
Cas9	Applied Biological Materials	Y300079 RRID:AB_2893376	Mouse IgG	Monoclonal	1:100*	1μg/mL
CD44	Ancell	193-020 RRID:AB_2893377	Mouse IgG	Monoclonal	1:250*	5μg/mL
Gephyrin	Synaptic Systems	147 RRID:AB_887716	Mouse IgG	Monoclonal	1:500*	1μg/mL
Gephyrin	Abcam	Ab32206 RRID:AB_2112628	Rabbit IgG	Polyclonal	1:500*	1μg/mL
HABP	Calbiochem	385911 RRID:AB_2861303	Bovine	Biotinylated	--	4μg/mL
HAS2	ThermoFisher Scientific	MA5-17087 RRID:AB_2538558	Mouse IgG	Monoclonal	1:200*	1μg/mL

Parvalbumin	Millipore	MAB1572 RRID:AB_2174013	Mouse IgG	Monoclonal	1:1000*	N/A
PSD-95	Santa Cruz	sc-32291 RRID:AB_628113	Mouse IgG	Monoclonal	1:50*	N/A
Rac1 (for Western)	Cytoskeleton Inc.	ARC03-FS RRID:AB_2893379	Mouse IgG	Monoclonal	1:500	1μg/mL
Total Rac1	Proteinech	24072-1-AP RRID:AB_2879427	Rabbit IgG	Polyclonal	1:200*	1μg/mL
VGAT	Synaptic Systems	131 004 RRID:AB_887873	Guinea Pig IgG	Polyclonal	1:1000*	N/A
VGLUT1	Synaptic Systems	135 304 RRID:AB_2621384	Guinea Pig IgG	Polyclonal	1:1000*	N/A
N-Cadherin	Cell Signaling Technology	#14215 RRID:AB_2798427	Mouse IgG	Monoclonal	1:200	1μg/mL

***IHC**

S. Table S2. Secondary Antibodies *IHC

Secondary Antibody	Brand	Catalog Number	Host	Class	Dilution	Concentration Used
Anti-Guinea Pig Alexa Fluor® 568	Invitrogen™	A-11075 RRID:AB_141954	Goat IgG	Polyclonal	1:500*	4µg/mL
Anti-Guinea Pig Alexa Fluor® 647	Invitrogen™	A-21450 RRID:AB_141882	Goat IgG	Polyclonal	1:500*	4µg/mL
Anti-Mouse Alexa Fluor® 488	Invitrogen™	A-11001 RRID:AB_2534069	Goat IgG	Polyclonal	1:500*	4µg/mL
Anti-Mouse Alexa Fluor® 568	Invitrogen™	A-11004 RRID:AB_2534072	Goat IgG	Polyclonal	1:500*	4µg/mL
Anti-Mouse Alexa Fluor® 647	Invitrogen™	A-21235 RRID:AB_2535804	Goat IgG	Polyclonal	1:500*	4µg/mL
Anti-Mouse Alexa Fluor® 488 IgM	Invitrogen™	A-21042 RRID:AB_141357	Goat IgG	Polyclonal	1:500*	4µg/mL
Anti-Rabbit Alexa Fluor® 488	Invitrogen™	A-11008 RRID:AB_143165	Goat IgG	Polyclonal	1:500*	4µg/mL
Anti-Rabbit Alexa Fluor® 568	Invitrogen™	A-11011 RRID:AB_143157	Goat IgG	Polyclonal	1:500*	4µg/mL
Anti-Rabbit Alexa Fluor® 647	Invitrogen™	A-21245 RRID:AB_2535813	Goat IgG	Polyclonal	1:500*	4µg/mL
Streptavidin 568	Molecular Probes™	S11226 RRID:AB_2893381	N/A	N/A	1:1000	2µg/mL
Streptavidin 488	Molecular Probes™	S11223 RRID:AB_2893382	N/A	N/A	1:1000	2µg/mL
Donkey anti-Mouse IgG (H+L) Cross Adsorbed Secondary Antibody, HRP	ThermoFisher Scientific	SA1-100 RRID:AB_325993	Donkey-anti-mouse IgG	Polyclonal	1:2500	0.016µg/mL

***IHC**

S. Table S3. Primer Sequences

Target	Sequence	Reference
Gapdh	Forward: GAATTTGGCTACAGCAACAGG	NM_002046
	Reverse: AGTGAGGGTCTCTCTCTTCC	
Has1	Forward: GGTCATGTACACAGCCTTCA	NM_001523
	Reverse: GCAGGATACACAGTGGAAGTAG	
Has2	Forward: CGTCATGGTCTTCATGTCTCTC	NM_005328
	Reverse: CCCACCCAGCTTTGTTTATTG	
Has3	Forward: CAGTGGTCACGGGTTTCTT	NM_005329
	Reverse: GTCAGCAGGAAGAGGAGAATG	
CD44	Forward: GACCTCTGCAAGGCTTTCAA	NM_001202557
	Reverse: TCCGATGCTCAGAGCTTTCTC	
